# 动物水平的绿原酸通过Notch1信号通路调控非小细胞肺癌凋亡及机制研究

**DOI:** 10.3779/j.issn.1009-3419.2017.08.09

**Published:** 2017-08-20

**Authors:** 维 李, 旭 刘, 国倩 张, 琳琳 张

**Affiliations:** 1 300193 天津，天津中医药大学第一附属医院检验科 Department of Laboratory, First Affiliated Hospital of Tianjin University of Traditional Chinese Medicine, Tianjin 300193, China; 2 300052 天津，天津医科大学总医院肿瘤科 Department of Oncology, General Hospital of Tianjin Medical University, Tianjin 300052, China

**Keywords:** 肺肿瘤, 肺癌细胞A549, 绿原酸, Notch1信号通路, PI3K/AKT通路, Lung neoplsms, Lung cancer A549 cell, Chlorogenic acid, Notch signaling pathway, PI3K/AKT pathway

## Abstract

**背景与目的:**

绿原酸类物质可通过调节细胞周期、诱导凋亡、抑制细胞生长等途径产生抗癌作用，Notch信号通路与人类许多肿瘤都存在密切的关系，本研究旨在探讨绿原酸通过Notch1信号通路控制非小细胞肺癌细胞凋亡的作用机制，为临床应用以及Notch1靶向药物的研究提供依据。

**方法:**

MTT法检测不同浓度的绿原酸对非小细胞肺癌细胞系A549细胞形态和细胞增殖的影响；流式细胞仪检测绿原酸对A549细胞的凋亡和细胞周期的影响；建立A549细胞的裸鼠荷瘤模型；测量肿瘤大小和重量；实时荧光定量PCR法检测Notch信号通路相关因子的mRNA表达水平；免疫印迹法检测Notch信号通路相关因子的蛋白表达水平。

**结果:**

绿原酸抑制A549细胞增殖，诱导A549细胞凋亡，增加细胞G_2_期/M期细胞百分比增加（*P* < 0.05），并且呈现剂量依赖趋势。在A549细胞的裸鼠荷瘤模型中，实验组肿瘤大小和体积明显小于对照组，差异具有统计学意义（*P* < 0.01）。试验组Notch1、VEGF、Delta4、HES1、HEY1 mRNA表达量较对照组明显减少（*P* < 0.05）。实验组Notch1蛋白明显减少，PTEN、p-PTEN、p-AKT明显增加（*P* < 0.05）。

**结论:**

在动物水平，绿原酸可能通过Notch1信号通路调控非小细胞肺癌的凋亡，可能是通过减少VEGF的表达，下调Delta 4水平，从而抑制Notch1信号通路的活化。Notch1信号通路可能通过PTEN与PI3K/AKT通路存在交叉调控作用。

## Abstract (en)

**Background and objective:**

It has been proven that chlorogenic acids can produce anticancer effects by regulating cell cycle, inducing apoptosis, inhibiting cell growth, Notch signaling pathways are closely related to many human tumors. The aim of this study is to study the mechanism of chlorogenic acid on apoptosis of nonsmall lung cancer through Notch1 pathway in animal level, and hope to provide theory basis on clinical treatment and research aimed at targeting Notch1 signaling in non-small cell carcinoma (NSCLC).

**Methods:**

MTT assay was used to evaluate the A549 cell proliferation under the treatment of chlorogenic acid. The effect of chlorogenic acid on apoptotic and cell cycle were detected by flow cytometry. The animal model of A549 cell transplanted in nude was established, tumer size and weight were detected. The mRNA level of Notch1 signal pathway related facter were detected by RT-PCR; the expression of Notch1 signal pathway related facter in tumor tissue was detected by western blot.

**Results:**

Chlorogenic acid inhibited the A549 cell proliferation. incresed cell apoptotic and cell percentagein G_2_/M (*P* < 0.05), and in a dose-dependent manner. In animal model, tumer size and weight were lower than control group, the difference was statistically significant (*P* < 0.05). The relative expression of mRNA of Notch1, VEGF, Delta4, HES1 and HEY1 were decreaced (*P* < 0.05) in tumor tissue which treated with chlorogenic. The expression of Notch1 were decreaced, PTEN, p-PTEN, p-AKT were increced significantly in tumor tissue which treated with chlorogenic (*P* < 0.05).

**Conclusion:**

Chlorogenic acid can regulate theapoptosis of non-small lung cancer through Notch pathway in animal level, which may be associated with the down-regulating the expression of VEGF and Delta4. Notch pathway may cross talk with PI3K/AKT pathway through PTEN in NSCLC.

肺癌是严重危害人类健康的恶性肿瘤之一，无论其发病率还是死亡率均居恶性肿瘤之首，已经成为严重危害人类生命健康的常见疾病。非小细胞肺癌（non-small cell lung cancer, NSCLC）是肺癌的一种，其患者数量为肺癌患者总数的80%以上^[1]^。目前NSCLC的治疗措施主要有手术、放疗、化疗及生物治疗等，早期手术可以治愈NSCLC，但大部分NSCLC患者确诊时已属晚期，目前尚无治愈的根本疗法。研究^[2]^表明，血管内皮生长因子（vascular endothelial growth factor, VEGF）及Notch信号通路，对肿瘤的形成和发展具有重要作用。Notch信号通路高度保守，由Notch受体、Notch配体、CSL DNA结合蛋白（CBF-1, suppressor of hairless, Lag）、其他的效应物和Notch的调节分子等组成。当相邻细胞的Notch受体、配体相互接触时，γ-分泌酶催化裂解形成Notch胞内段，之后Notch胞内段进入细胞核内结合并激活转录因子RBP-Jk，调控转录因子超级组成员Hes和Hey分子的转录，从而调节细胞的分化、发育、增殖、凋亡等过程。Notch通路还与其他信号通路存在交叉调控，从而扩大其生物调节作用^[3]^。目前已有不少研究Notch通路与肺癌的关系^[4, 5]^，可是尚未形成一个明确的结论，而Notch通路生物学作用下的具体的机制更是十分缺乏，仍然有待进一步研究。

近几年来，中药活性成分的抑癌作用得到了广泛关注，在诱导细胞凋亡方面的作用具有重要意义。绿原酸（chlorogenic acid, CGA）由奎尼酸（quinic acid, QA）与反式肉桂酸（trans cinnamic acidst-CA）缩合而成的酯类化合物家族，是多种中草药中的一种活性成分，具有抗炎作用、抗病毒作用、降血脂和血糖作用、抗氧化作用、增强机体免疫力等诸多生物学活性。已有研究^[6]^证明，绿原酸具有抑制肺癌细胞增值和转移，诱导肺癌细胞凋亡的作用。本研究建立了NSCLC细胞裸鼠模型，通过检测Notch信号通路及相互作用信号通路的关键蛋白的表达，进一步阐明绿原酸通过Notch1信号通路调控NSCLC凋亡的作用机制。

## 1 材料和方法

### 1.1 药品和试剂

绿原酸（250 mg, > 98%）购自Sigma-Aldrich公司（溶解于1640培养基，0.22 μm滤膜过滤），胎牛血清购自上海沪峰化工有限公司，RPMI-1640培养液购自上海博麦德生物技术有限公司，胰蛋白酶购自GIBCO公司，Annexin V-FITC细胞凋亡检测试剂盒购自碧云天生物技术研究所，TRizol购自北京百奥森泰生物科技有限公司，总蛋白提取试剂盒购自Sigma-Aldrich公司，BCA蛋白浓度测定试剂盒购自上海美吉生物医药科技有限公司，反转录试剂盒、SYBR Green Mix、DNA Marker购自北京全式金生物科技有限公司，PTEN抗体（兔抗）、p-PTEN抗体（p-Ser370，兔抗）、p-Akt（p-Ser473，兔抗）抗体购自Santa Cruz公司，Notch1抗体（兔抗）、GAPDH抗体（兔抗）、HRP-二抗（羊抗兔）等相关抗体购自Exapha Biologicals公司，其他试剂购自北京鼎国昌盛生物试剂公司。引物由上海英骏生物技术公司合成。

### 1.2 主要设备

多功能酶标仪（Wallac公司）；TS-100倒置相差显微镜购自日本Nikon公司；Real-time PCR仪购自BIO-RAD公司，二氧化碳培养箱（RCO3000TVBA）购自美国REVCO公司。

### 1.3 实验动物

实验用裸鼠BALB/c-nu由北京华阜康生物科技有限公司提供许可证号：[SCXK（京）2014-0004]，均为4周龄的健康SPF级雄性小鼠，体重为（18.77±1.02）g，裸鼠在无特定病原体条件下的层流架内饲养，无菌操作下定期更换笼具、垫料、饮用水和标准饲料。

### 1.4 细胞培养

NSCLC细胞株A549购自中国医学科学院中国协和医科大学细胞库，细胞培养液为含10%胎牛血清RPMI-1640，其中含终浓度为100 μg/mL青霉素和链霉素，2 mmol/L的L-谷氨酰胺；将细胞置于37 ℃、5% CO_2_孵箱中培养；2 d传代一次，取对数生长期细胞用于实验。

### 1.5 MTT法检测细胞增殖抑制率

将对数生长期A549细胞接种于96孔板中，每孔接种1×10^5^个细胞；培养24 h之后，在培养基加入梯度浓度的绿原酸（终浓度为：0、10 μg/mL、50 μg/mL、100 μg/mL），每个浓度设6个平行孔，绿原酸终浓度为0 μg/mL的为对照组；分别在药物处理A549细胞24 h、48 h、72 h后，每孔加入20 μL的5 mg/mL的MTT试剂，置于细胞培养箱中孵育4 h后吸出，加入150 μL的DMSO试剂，充分震荡后，用多功能酶标仪在490 nm波长下测定吸光度。计算细胞增殖抑制率。抑制率（%）=（1-药物处理组OD值/对照组OD值）×100%。

### 1.6 流式细胞仪检测细胞凋亡检测和细胞周期分析

将对数生长期A549细胞接种于6孔板，调节细胞密度，2 mL/孔，每孔细胞数目大约为4×10^5^个。24 h后加入系列浓度梯度的绿原酸培养基，使绿原酸的终浓度为：0、10 μg/mL、50 μg/mL、100 μg/mL；常规培养72 h后收集细胞，用195 μL的Bind Buffer重悬细胞，加入5 μL Annexin V-FITC混匀，室温避光孵育10 min。离心去上清后，用190 μL的Bind Buffer重悬细胞，再加入10 μL碘化丙啶（100 mg/L），并与避光放置，随即进行流式细胞仪检测细胞凋亡情况，并用软件进行数据分析。另一组细胞采用梯度绿原酸处理细胞72 h后收集细胞，PBS清洗后用预冷的95%乙醇处理，离心去上清后，碘化丙啶（100 mg/L）（含RNase 1 g/L）0.5 mL，室温避光孵育30 min，随即进行流式细胞仪进行细胞周期分析。

### 1.7 建立裸鼠成瘤模型

大量培养A549肺癌细胞，将生长状态良好的细胞消化后计数，将2×10^6^的肿瘤细胞种植于裸鼠皮下，共接种30只裸鼠，饲养观察裸鼠成瘤情况。在接种2周-3周后，肿瘤大小约1 cm^3^时，选择肿瘤大小接近的20只小鼠，根据随机分层法分组，分为2组。实验组每天在肿瘤区域注射绿原酸溶液（100 μg/mL）；对照组每天注射相同体积的生理盐水；每次注射体积为100 μL。连续观察并测量肿瘤的生长情况：每周测肿瘤长径（L），短径（W）。肿瘤大小按公式V=1/2LW^2^计算。

给药4周后处死裸鼠取瘤称重，检查肝脏肺部肿瘤的转移情况。每个肿瘤取两部分，一部分用于提取瘤组织蛋白，另一部分提取组织RNA。

### 1.8 实时荧光定量PCR（Real-time PCR）

检测mRNA相对含量取100 mg左右肿瘤组织，加入1 mL TRIzol，用研钵研磨至无明显肉眼可见固体。按照说明书操作步骤，提取RNA。然后按照反转录试剂盒步骤，将提取的RNA进行反转录，得到的cDNA用SYBR Green染料结合法，进行Real-time PCR。目的基因的相对表达量经过内参标化后分析（表 1）。

**1:** 相关基因PCR引物 Related gene PCR primers

Gene name	5'-3'	3'-5'
*GAPDH*	GAGTCAACGGATTTGGTCGT	TTGATTTTGGAGGGATCTCG
*Delta 4*	GCAGAACTTACATCACCTCA	GCATTGCTGCCTCTAGTTAT
*VEGF*	TCGGGCCTCCGAAACCATGA	CCTGGTGAGAGATCTGGTTC
*Notchl*	ACCTCTTTGGGCTGGTATTG	AACGGACAGCTTTGGATTTC
*HES1*	TGAAGAAAGATAGCTCGCGG	GGTACTTCCCCAGCACACTT
*HEY1*	TGGATCACCTGAAAATGCTG	CGAAATCCCAAACTCCGATA

### 1.9 免疫印迹（Western blot）检测蛋白表达

取少量肿瘤组织，按照总蛋白提取试剂盒步骤，提取组织总蛋白，用BCA法进行蛋白浓度测定（具体步骤见商品说明书）。将蛋白液与蛋白上样缓冲液混合后，100 ℃变性10 min，然后进行SDS-聚丙烯酰胺凝胶电泳。电泳后，取下凝胶，标记方向后进行转膜，封闭，孵育一抗（兔源Notch1、p-PTEN、PTEN、p-Akt抗体按照1:800浓度稀释比稀释，GAPDH抗体按照1:2, 000浓度比稀释），孵育二抗（羊抗兔二抗按照1:5, 000浓度稀释比稀释），最后经过充分洗膜后，进行曝光，分析结果。

### 1.10 统计学分析

采用SPSS 18.0统计学软件进行数据分析，数据均以均数±标准差（Mean±SD）表示，组间比较采用Student’s *t*检验，*P* < 0.05表示差异具有统计学意义。

## 2 结果

### 2.1 绿原酸对NSCLC

A549细胞增殖的影响绿原酸处理A549细胞48 h后，对照组细胞生长状态较好；而给药组细胞随着绿原酸剂量的升高，细胞脱落悬浮，破碎增多，部分细胞呈凋亡状态。

在绿原酸处理A549细胞24 h、48 h、72 h后，分别采用MTT法检测细胞增殖的影响。结果显示，与对照组比较，绿原酸对A549细胞的抑制率明显升高（*P* < 0.05），并且呈现剂量依赖趋势。在100 μg/mL绿原酸作用在72 h时，抑制率达最高（61.63%）（表 2）。

**2:** 绿原酸对A549细胞增殖的抑制作用（Mean±SD） Inhibitory effect of chlorogenic acid on proliferation of A549 cells (Mean±SD)

Group	CGA(*μ*g/mL)	24 h			48 h			72 h	
		OD_490_	Inhibitory rate		OD_490_	Inhibitory rate		OD_490_	Inhibitory rate
NC	0	0.75±0.02			0.81±0.03			0.86±0.03	
Experimental	10	0.70±0.05^*^	6.67%		0.71±0.05^**^	12.35%		0.68±0.07^**^	20.93%
group	50	0.59±0.04^**^	21.33%		0.61±0.07^**^	24.69%		0.52±0.05^**^	39.53%
	100	0.44±0.03^**^	41.33%		0.45±0.08^**^	44.44%		0.33±0.09^**^	61.63%
The experimental group compared with the control group, ^*^P < 0.05, ^**^P < 0.01.									

### 2.2 绿原酸对NSCLC

A549细胞凋亡和细胞周期的影响流式细胞仪检测绿原酸作用于A549细胞72 h后，随着绿原酸浓度增加，G_2_期/M期细胞百分比逐渐增加，且均高于对照组，差异显著（*P* < 0.05或*P* < 0.01）；随着给药浓度增加，细胞的凋亡率逐渐增加，均高于对照组，差异显著（*P* < 0.05或*P* < 0.01）（图 1，表 3）。

**1:**
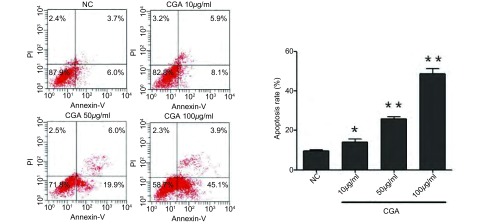
绿原酸对A549细胞凋亡率的影响。实验组与对照组比较，^*^P < 0.05，^**^P < 0.01。 Effect of chlorogenic acid on apoptosis rate of A549 cells. The experimental group compared with the control group, ^*^P < 0.05, ^**^P < 0.01.

**3:** 绿原酸对A549细胞周期分布时相的影响（Mean±SD） Effect of chlorogenic acid on the cell cycle profile of A549 cells (Mean±SD)

Group	CGA (*μ*g/mL)	Cell cycle		
		G_0_/G_1_	S	G_2_/M
NC	0	66.7±0.69	25.9±0.60	7.2±0.74
Experimental group	10	57.1±0.72	28.9±0.61	14.6±0.65^*^
	50	47.9±0.61	31.0±0.32	21.0±0.36^**^
	100	41.1±0.62	28.0±0.63	30.7±0.57^**^
The experimental group compared with the control group, ^*^P < 0.05, ^**^P < 0.01.				

### 2.3 绿原酸对A549细胞裸鼠荷瘤模型肿瘤增殖的影响

通过A549细胞裸鼠荷瘤模型发现，绿原酸能够抑制裸鼠肿瘤的生长增殖，实验组裸鼠肿瘤大小明显小于对照组，具有统计学差异（*P* < 0.05），见表 4、图 2、图 3。将裸鼠处死后，发现试验组裸鼠无转移瘤出现，而在对照组肺中发现有6例微小转移瘤。实验组肿瘤重量为（0.48±0.12）g，对照组为（0.83±0.22）g，两组比较差异具有统计学意义（*P* < 0.01）。

**4:** 不同时间点裸鼠肿瘤大小的比较（Mean±SD, cm^3^） Comparison of tumor size in nude mice at different time points (Mean±SD, cm^3^)

Time	NC	Experimental group	*P*
1^st^ week	1.671±0.124	1.222±0.048^*^	< 0.05
2^nd^ week	1.960±0.171	1.401±0.078^*^	< 0.05
3^rd^ week	2.196±0.132	1.541±0.098^**^	< 0.01
4^th^ week	2.472±0.118	1.706±0.112^**^	< 0.01
The experimental group compared with the control group, ^*^P < 0.05, ^**^P < 0.01.			

**2:**
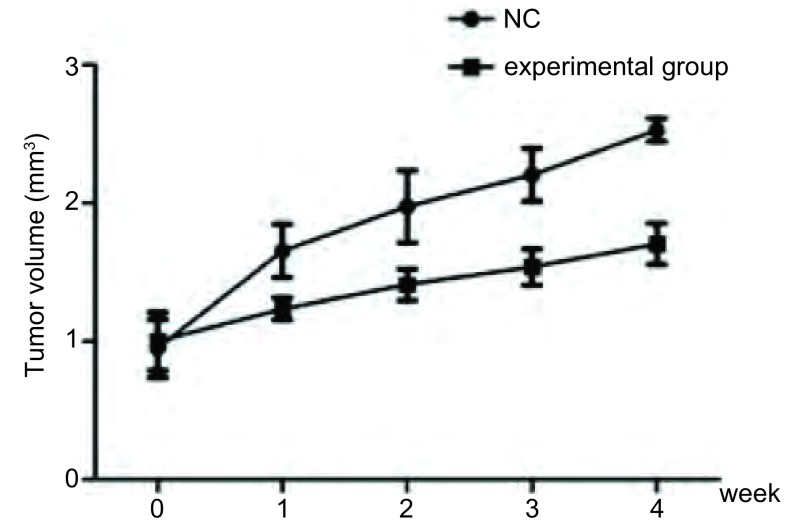
绿原酸对肿瘤生长增殖的影响 Effect of chlorogenic acid on tumor growth and proliferation

**3:**
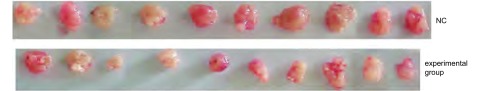
两组裸鼠肿瘤比较 Comparison of nude mice tumers of two groups

### 2.4 绿原酸对Notch信号通路相关分子mRNA表达量的影响

实验组Notch1的配体Delta4、VEGF的mRNA相对表达量明显小于与对照组（*P* < 0.05）。实验组Notch1信号通路下游效应分子HES1、HEY1的mRNA相对表达量明显降低（*P* < 0.05）。见图 4。

**4:**
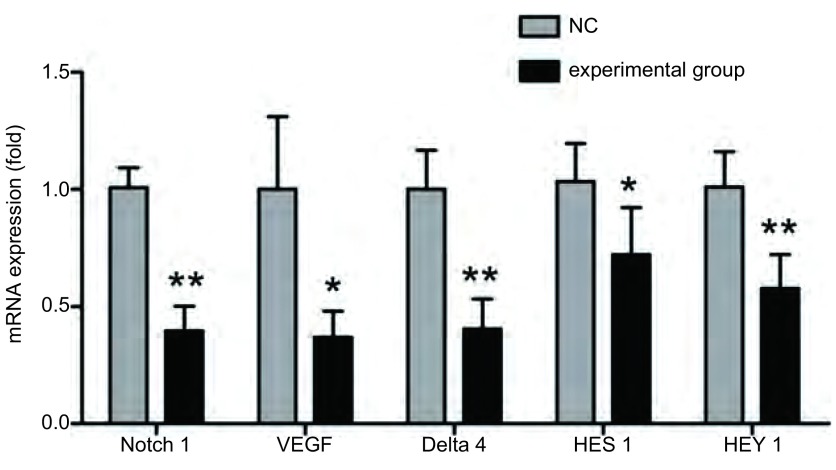
Real-time PCR检测Delta4、VEGF、Notch1、HES1、HEY1的mRNA表达水平。实验组与对照组比较，^**^P < 0.01，^*^P < 0.05。 mRNA expression of Delta4, VEGF, Notch1, HES1, HEY1 detected by Real-time PCR. The experimental group compared with the control group, ^**^P < 0.01, ^*^P < 0.05.

### 2.5 绿原酸对PTEN-PI3K/AKT通路相关因子蛋白表达的影响

检测Notch1及PTEN-PI3K/AKT通路相关因子的蛋白表达水平，结果显示，与对照组比较，试验组Notch1蛋白表达明显降低，且p-PTEN、PTEN蛋白表达量升高，总的PTEN蛋白表达量增多；试验组p-Akt的表达量减少。见图 5。

**5:**
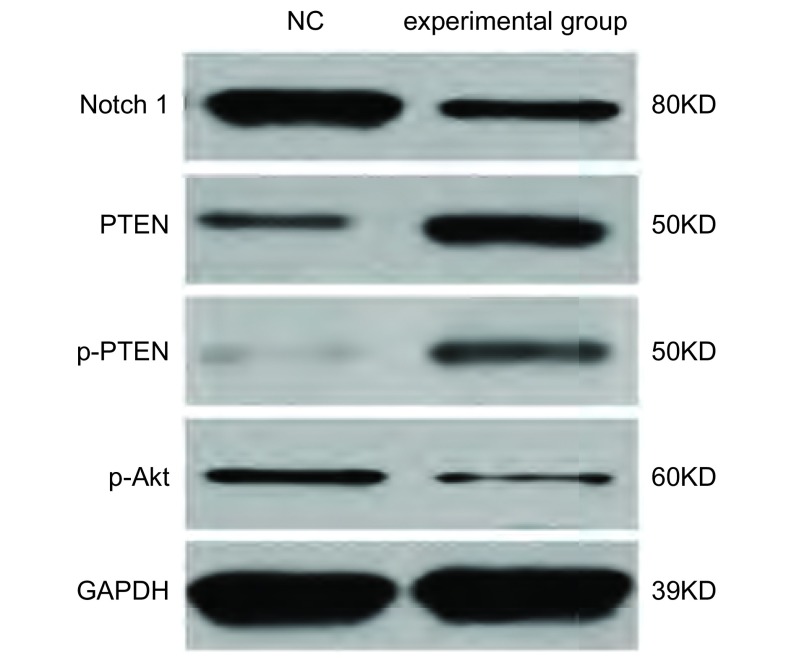
Western blot检测Notch1、PTEN、p-PTEN、p-Akt蛋白表达水平 Expression of Notch1, PTEN, p-PTEN, p-Akt protein detected by Western blot

## 3 讨论

绿原酸类物质是植物体中重要的次生代谢产物，广泛存在于高等双子叶植物和蕨类植物中，杜仲、金银花、咖啡等植物中绿原酸类物质含量较高。绿原酸类物质可通过调节细胞周期、诱导凋亡、抑制细胞生长等途径产生抗癌作用，对肺癌、乳腺癌、肝癌具有显著的抑制作用，被认为是癌症的有效化学防护剂^[6-8]^。但绿原酸对肺癌的抑制作用机制的研究并不多见。

Notch信号通路与人类许多肿瘤都存在密切的关系。Notch信号的产生是通过相邻细胞的Notch配体与受体相互作用，Notch蛋白经过三次剪切，由胞内段释放入胞质，激活HES、HEY、HERP等碱性-螺旋-环-螺旋（bHLH）转录抑制因子家族的靶基因，发挥生物学作用。Notch信号通路不仅在组织器官的正常发育中起作用，还与一些肿瘤的发生、发展密切相关^[9-13]^。有研究表明Notch信号通路在肺癌的发生发展起着重要作用^[14]^，但是具体的作用机制一直没有达成共识。本文通过研究Notch受体、配体以及相关因子在非小细胞肺癌细胞裸鼠荷瘤模型中的表达，探讨Notch1信号通路与肺癌发生发展的关系，阐明绿原酸抑制非小细胞肺癌细胞凋亡的原理，同时为以Notch1信号通路为靶点的治疗提供分子机制方面的理论支持。

本研究发现，在细胞及动物水平上，绿原酸能有效抑制非小细胞肺癌细胞的生长增殖，促进细胞的凋亡。同时本研究发现实验组肺癌组织的Notch1 mRNA的平均表达水平显著下降，同时下调下游的HES1和HEY1 mRNA的表达，提示绿原酸可以通过Notch1信号通路转录水平调控非小细胞肺癌凋亡的。有研究^[15]^发现，抑制VEGF表达可以达到抑制人非小细胞肺癌A549细胞裸鼠移植瘤的血管生成的作用。VEGF可诱导Notch1配体Delta4表达增高，从而可启动Notch信号传导通路。研究发现Notch信号通路配体Delta4以及VEGF的表达水平，发现实验组减少Delta4以及VEGF的mRNA水平的表达。因此，推测绿原酸可能是通过减少VEGF的表达，下调Delta4水平，从而抑制Notch1信号通路的活化。

Notch信号通路不仅能够直接调控大量的基因表达，而且还可以与其他信号通路（包括TGF-β、NF-κB、Hif-1α等）相互作用而扩大其调控范围^[16-18]^。近年来，Notch通路与PI3K-AKT等通路之间的互相作用受到广泛的关注抑癌基因PTEN在PI3K-AKT信号通路中发挥着至关重要的作用，PTEN的失活会导致PI3K-AKT通路活化，PTEN能够使磷脂酰肌醇（3, 4, 5）-三磷酸（PIP3）脱磷酸，使PIP3磷酸化呈低水平，抑制AKT的活化，从而下调PI3K/AKT通路相关因子表达。在PI3K-AKT通路中，PTEN有时候以磷酸化形式（p-PTEN）出现，继而启动下游级联反应，导致一系列生物学行为的发生。AKT磷酸化激活下游信号因子活化。本研究检测Notch1信号通路与PTEN-PI3K/AKT通路的相互作用是否存在交叉调控，结果发现，试验组的p-PTEN、PTEN蛋白表达量都出现了一定程度的升高，总的PTEN蛋白表达量增多；与对照组比较，试验组p-Akt的表达量减少。提示Notch1通路可能通过对抑癌基因PTEN的调控来影响肺癌细胞的生物学功能，通过PTEN与PI3K/AKT通路存在交叉调控作用。

Notch信号通路非常复杂，且调控机制目前仍未清楚阐明。Notch信号通路在肿瘤的发生发展中非常重要，但是目前该通路的研究以及Notch信号通路与其他信号通路的交叉调控方面的研究相对较少，希望能后续进一步详细阐明Notch信号通路的生物学作用机制，填补这一块研究的空白，为肺癌的靶向治疗提供更有力的理论基础。

## References

[1] Pisters KM, Chevalier TL (2005). Adjuvant chemotherapy in completely resectednon-small-cell lung cancer. J Clin.

[2] Balint K, Xiao M, Pinnix CC (2005). Activation of Notch1 signaling is required for beta-catenin-mediated human primary melanoma progression. J Clin Invest.

[3] Mungamuri SK, Yang X, Thor AD (2006). Survival signaling by Notch1: mammalian target of Rapamycin (mTOR)-dependent inhibition of p53. Cancer Res.

[4] Zheng YF, Zheng ZH (2006). Notch and Ras/MAPK signaling pathways in embryonic development and tumor occurrence. Yi Xue Fen Zi Sheng Wu Xue Za Zhi.

[5] Sriuranpong V, Borges MW, Ravi RK (2001). Notch signaling induces cell cycle arrest in small cell lung cancer cells. Cancer Res.

[6] Tian W, Dou YW, Wang HT (2016). Study on apoptosis of lung cancer cells induced by chlorogenic acid and its mechanism. Jie Fang Jun Yu Fang Yi Xue Za Zhi.

[7] Ye XL, Liu Y, Qiu G (2012). Inhibitory effect of chlorogenic acid on mouse EMT-6 breast cancer. Zhong Yao Yao Li Yu Lin Chuang.

[8] Lai LL, Xiao Y, Peng XF (2017). Effects of chlorogenic acid extract from eucommia ulmoides oliv. leaves combined with crocin on cholesterol metabolism in hepatocellular carcinoma cells. Shandong Yi Yao.

[9] Artavanistsakonas S, Rand MD, Lake RJ (1999). Notch signaling: cell fate control and signal integration in development. Science.

[10] Deftos ML, Bevan MJ (2000). Notch signaling in T cell development. Curr Opin Immunol.

[11] Maillard I, Pear WS (2003). Notch and cancer: Best to avoid the ups and downs. Cancer Cell.

[12] Balint K, Xiao M, Pinnix CC (2005). Activation of Notch l signaling is required for beta-caten in-mediated human primar melanom a progression. J Clin Invest.

[13] Weng AP, Aster JC (2004). Activating mutations of NOTCH1 in human T cell acute lymphoblastic leukemia. Science.

[14] Collins BJ, Kleeberger W, Ball DW (2004). Notch in lung development and lung cancer. Semin Cancer Biol.

[15] Jiang P, Cen HF, Mao Y (2011). Study on Notch signaling pathway and expression of VEGF protein in non-small cell lung cancer. Zhongguo Lin Chuang Yao Li Za Zhi.

[16] Palomero T, Wei KL, Odom DT (2006). NOTCH1 directly regulates c-MYC and activates a feed-forward-loop transcriptional network promoting leukemic cell growth. Proc Natl Acad Sci U S A.

[17] Poellinger L, Lendahl U (2008). Modulating Notch signaling by pathway-intrinsic and pathway-extrinsic mechanisms. Curr Opin Genet Dev.

[18] Samon JB, Champhekar A, Minter LM (2008). Notch1 and TGF beta1 cooperatively regulate Foxp3 expression and the maintenance of peripheral regulatory T cells. Blood.

